# CaMKII, but not protein kinase A, regulates Rpt6 phosphorylation and proteasome activity during the formation of long-term memories

**DOI:** 10.3389/fnbeh.2013.00115

**Published:** 2013-08-30

**Authors:** Timothy J. Jarome, Janine L. Kwapis, Wendy L. Ruenzel, Fred J. Helmstetter

**Affiliations:** Department of Psychology, University of Wisconsin-MilwaukeeMilwaukee, WI, USA

**Keywords:** fear conditioning, protein degradation, synaptic plasticity, memory

## Abstract

CaMKII and Protein Kinase A (PKA) are thought to be critical for synaptic plasticity and memory formation through their regulation of protein synthesis. Consistent with this, numerous studies have reported that CaMKII, PKA and protein synthesis are critical for long-term memory formation. Recently, we found that protein degradation through the ubiquitin-proteasome system is also critical for long-term memory formation in the amygdala. However, the mechanism by which ubiquitin-proteasome activity is regulated during memory formation and how protein degradation interacts with known intracellular signaling pathways important for learning remain unknown. Recently, evidence has emerged suggesting that both CaMKII and PKA are capable of regulating proteasome activity *in vitro* through the phosphorylation of proteasome regulatory subunit Rpt6 at Serine-120, though whether they regulate Rpt6 phosphorylation and proteasome function *in vivo* remains unknown. In the present study we demonstrate for the first time that fear conditioning transiently modifies a proteasome regulatory subunit and proteasome catalytic activity in the mammalian brain in a CaMKII-dependent manner. We found increases in the phosphorylation of proteasome ATPase subunit Rpt6 at Serine-120 and an enhancement in proteasome activity in the amygdala following fear conditioning. Pharmacological manipulation of CaMKII, but not PKA, *in vivo* significantly reduced both the learning-induced increase in Rpt6 Serine-120 phosphorylation and the increase in proteasome activity without directly affecting protein polyubiquitination levels. These results indicate a novel role for CaMKII in memory formation through its regulation of protein degradation and suggest that CaMKII regulates Rpt6 phosphorylation and proteasome function both *in vitro* and *in vivo*.

## Introduction

The formation of long-term fear memories requires transient increases in the activity of a number of intracellular signaling pathways which regulate protein synthesis in the amygdala (Johansen et al., 2011). Of these pathways, protein kinase A (PKA) and Calcium-calmodulin-dependent protein kinase II (CaMKII) have received considerable attention as primary regulators of long-term memory formation and stability at amygdala synapses (Mayford et al., 1996; Abel et al., 1997; Schafe and LeDoux, 2000; Bejar et al., 2002; Moita et al., 2002; Rodrigues et al., 2004; Tronson et al., 2006). These protein kinases are thought to be critical for memory formation by regulating transcription through phosphorylation of the cre-response element binding (CREB) protein and consequent increases in *de novo* protein synthesis (Johansen et al., 2011). This suggests that memory impairments observed following genetic and pharmacological manipulations of CaMKII and PKA signaling could occur due to disrupted downstream signaling necessary for the well described transcriptional and translational processes thought to be important for normal memory formation in the amygdala (Bailey et al., 1999; Parsons et al., 2006). However, an alternate hypothesis is that CaMKII and PKA also regulate protein degradation during memory formation (Jarome and Helmstetter, 2013).

Consistent with this, evidence has emerged suggesting that both CaMKII and PKA can regulate increases in ubiquitin-proteasome mediated protein degradation *in vitro* through their regulation of the proteasome complex (Zhang et al., 2007; Djakovic et al., 2009). For example, CaMKII acts as a scaffold to recruit proteasomes to dendritic spines in an activity-dependent manner, where it then can regulate increases in proteasome activity (Bingol et al., 2010). Interestingly, both CaMKII and PKA have been shown to phosphorylate the proteasome regulatory subunit Rpt6 at Serine-120, a site known to be critical for the regulation of increases in proteasome activity and activity-dependent changes in synaptic strength and new dendritic spine growth (Djakovic et al., 2012; Hamilton et al., 2012). However, it is currently unknown whether CaMKII and PKA regulate Rpt6-S120 phosphorylation and proteasome activity *in vivo* to support learning.

Recently, we have identified protein degradation as a critical step in long-term memory formation in the amygdala (Jarome et al., 2011). Consistent with this, several studies have demonstrated a role for protein degradation during long-term memory formation (Lopez-Salon et al., 2001; Yeh et al., 2006; Artinian et al., 2008; Rodriguez-Ortiz et al., 2011; Felsenberg et al., 2012). However, the molecular mechanisms that control proteasome activity during this consolidation period are currently unknown. One possibility is that proteasome activity is increased following fear conditioning through CaMKII- or PKA-mediated phosphorylation of Rpt6-S120. However, to date no study has directly examined if Rpt6-S120 phosphorylation and proteasome activity are increased following learning and if CaMKII and PKA regulate protein degradation *in vivo*. We tested this idea by measuring Rpt6-S120 phosphorylation and *in vitro* proteasome activity in the amygdala of fear conditioned animals following *in vivo* manipulation of CaMKII and PKA signaling. Our results demonstrate for the first time that CaMKII and PKA play dissociable roles in regulating protein degradation during memory formation.

## Materials and methods

### Subjects

Male Long Evans rats weighing between 300 and 350 g (~3-months old) at time of arrival were obtained from Harlan (Madison, WI). All animals were housed individually in shoebox cages with free access to water and rat chow throughout the duration of the experiment (3, 4 weeks). The colony room was maintained under a 14:10-h light/dark cycle. Experiments took placed during the light portion of the cycle. All procedures were approved by the University of Wisconsin-Milwaukee Institutional Animal Care and Use Committee and conducted within the ethical guidelines of the National Institutes of Health.

### Surgery

All animals were anesthetized with 2–4% isoflurane in 100% O_2_ and implanted with bilateral stainless steel 26-gauge cannulae aimed at the basolateral nucleus of the amygdala (BLA) using stereotaxic coordinates (AP −3.0 mm, ML+/−5.0 mm, DV −7.2 mm) relative to bregma. Cannulae were secured to the skull with stainless steel screws, superglue, and dental acrylic. Rats were given a recovery period of at least 7 d before behavioral testing. Since all brain tissue was collected for western blot analysis, histology could not be performed. As a result, no animals could be excluded for misplaced cannula.

### Apparatus

Auditory fear conditioning was conducted in a set of four Plexiglas and stainless-steel observation chambers (Context A; internal dimensions: 21 × 28 × 21 cm) housed in sound-attenuating chambers. The floor was comprised of 18 stainless steel bars 5 mm in diameter spaced 12 mm apart and connected to a shock generator. Ventilation fans produced 62–64 dB of background noise. Each chamber was equipped with a speaker centered in the middle of one end of the chamber. Before testing of each animal, Context A was cleaned with a 5% ammonium hydroxide solution.

### Drug preparation and infusion procedure

In all cases, rats received bilateral infusions into the amygdala. The total volume of the infusion (0.5 μl/side) was given over 60 s, and the injection cannula remained in place an additional 90 s to ensure diffusion away from the injector tip. The injection cannulae were cut to extend ~0.5 mm beyond the guide cannula. Rats were returned to their home cages after infusions. The specific PKA inhibitor myristoylated Protein Kinase Inhibitor 14–22 amide (myr-PKI, 4 μg/μl; EMD Biosciences, Billerica, MA) or specific CaMKII inhibitor myristoylated autocamtide-2 related inhibitory peptide (myr-AIP, 6 ng/μl; Enzo Life Sciences, Farmingdale, NY) were dissolved in distilled H_2_O. The myristolated versions of these peptides were used to enhance cell permeability and are highly specific to PKA or CaMKII (Glass et al., 1989; Ishida et al., 1995). These dosages were determined based on prior research (Ouyang et al., 2008; Ma et al., 2009; Tinsley et al., 2009). The myr-PKI peptide has been shown to impair fear memory formation in the hippocampus to a similar degree as the more common PKA inhibitor Rp-cAMPs (Ma et al., 2009). Additionally, while the broader CaMKII inhibitor KN62 has been shown to impair fear memory formation in the amygdala (Rodrigues et al., 2004), myr-AIP has been shown to be a more robust inhibitor of CaMKII activity and long-term memory formation than KN62 in other brain regions (Tinsley et al., 2009).

### Behavioral procedures

Animals were trained to auditory fear conditioning as described previously (Jarome et al., 2011, 2012). Briefly, following 3 days of acclimation to the transporting and injection procedures, animals were placed in novel Context A and after a 6 min baseline, presented with 4 pairings of a white noise (72 dB, 10 s) with a footshock (1.0 mA, 1 s), 90-s ITI (intertrial interval). After a 4 min postshock period, the animals were removed from the chambers. Mircoinfusions were given immediately following the completion of the training session. For associative control experiments, animals were exposed to the white noise alone or underwent an immediate shock procedure (SK) as described previously (Jarome et al., 2011). Briefly, in the immediate shock procedure, animals were placed in Context A and immediately received 4 presentations of the footshock (1.0 mA, 1 s, 1-s ITI). Animals were then removed from the chamber following the final shock presentation. Animals are thus exposed to the shock but are unable to form a context-shock association using parameters such as these. In the white noise only control (WN), animals received an identical training session as the normal trained group except that the shock presentations were omitted. During training days for the associative control experiment (Figure 2), each of the four conditions (homecage, SK, WN, and trained) were equally represented during every batch of tissue collection. Order of conditioning for the associative control experiment was Trained, WN, SK, and then repeated.

### Tissue collection

Animals were overdosed on isoflurane and the brain rapidly removed (<1 min) and immediately frozen on dry ice. Amygdala tissue was then dissected out by blocking the brain in a rat brain matrix (Harvard Apparatus, Holliston, MA) incubated on dry ice. Tissue samples were homogenized in lysis buffer (50 mM Tris-HCl, 6 mM sodium deoxycholate, 150 mM NaCl, 1 mM EDTA, 1 mM NaF, 1 μg/μl PMSF, 1 μg/μl leupeptin, 1 μg/μl aprotinin, 1% SDS, 1 mM sodium orthovanadate) and immediately placed on dry ice. Samples were stored at −80°C until needed. Samples were thawed and then centrifuged at 4000 rpm for 20 min at 4°C; the supernatant was removed and measured using a Bradford protein assay kit (BioRad, Hercules, CA).

### 20S proteasome activity assay

**Figure 1:**
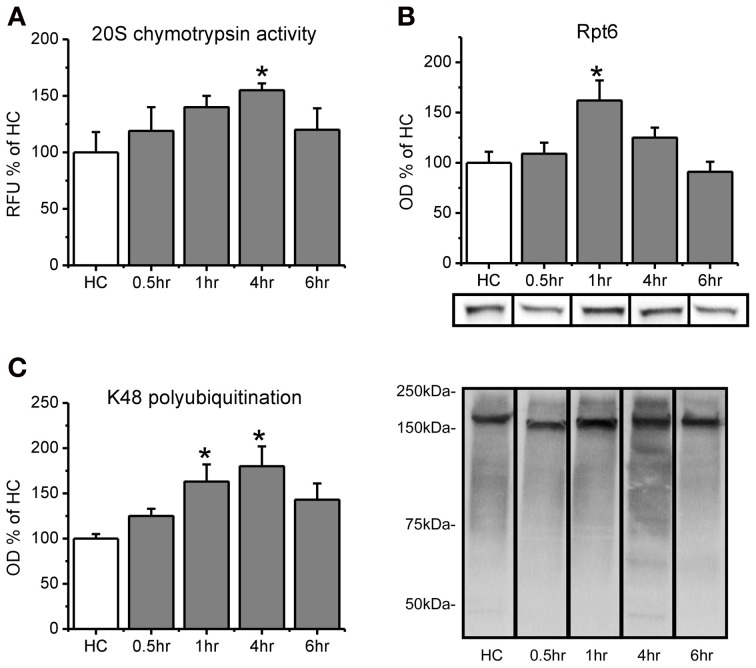
**Proteasome chymotrypsin-like activity is increased in the amygdala after fear conditioning.** Rats were trained to auditory fear conditioning (*n* = 10–11 per group) and amygdala tissue collected 0.5, 1, 4, or 6 h later for *in vitro* proteasome activity assay and Western blotting. **(A)** Amygdala lysates collected 4 h after fear conditioning showed enhanced degradation of the fluorogenic substrate LLVY-AMC relative to homecage (HC) controls. **(B)** There were transient increases in proteasome subunit Rpt6 that returned to baseline levels by 4 h. **(C)** Lysine-48 linked polyubiquitination was increased from 1–4 h after fear conditioning. Lower right panel shows representative K48 polyubiquitin blots for each group from the same gel. ^*^p < 0.05 from HC controls.

**Figure 2:**
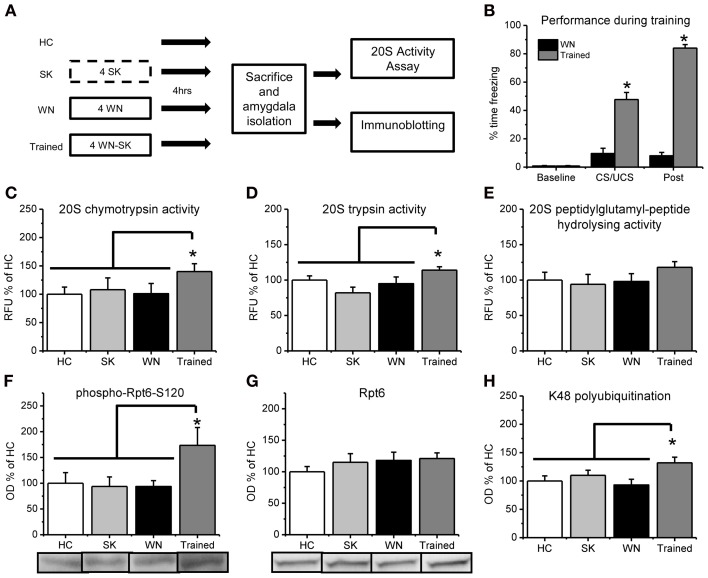
**Fear conditioning increases *in vitro* proteasome activity and phosphorylation of Rpt6-S120 in amygdala tissue. (A)** Rats were exposed to several pairings of an auditory cue with a footshock or exposed to the shock (SK) or white noise (WN) individually and amygdala tissue collected 4 h later (*n* = 9–11 per group). Dotted line denotes 10 s in the training context, while solid lines denote 15 min in the training context. **(B)** Animals that received pairings of the white noise with the footshock froze significantly more during training than animals receiving the WN alone. **(C–E)** Proteasome chymotrypsin-like **(C)** trypsin-like **(D)** and peptidylglutamyl-peptide hydrolyzing-like **(E)** activities were increased only in rats that received the auditory cue paired with the footshock. **(F)** Fear conditioning increased phosphorylation of the proteasome regulatory subunit Rpt6 at Serine120 **(G)** There were no increases in the Rpt6 subunit but **(H)** fear conditioned animals showed enhanced protein polyubiquitination. ^*^p < 0.05 from WN controls **(B)** or HC, SK and WN controls **(C–H)**.

Proteasome activity assays were performed as described previously with a small scale modification (Lopez-Salon et al., 2001; Ehlers, 2003; Upadhya et al., 2006). Samples (50μg, Figure 4; 100μg, Figures 1, 2) were diluted in DDH_2_O and mixed with reaction buffer (250 mM HEPES, pH 7.5, 5 mM EDTA, 0.5% NP-40, 0.01% SDS, 5 mM ATP). Fluorogenic peptides Suc-LLVY-AMC (Millipore, Billerica, MA), Bz-VGR-AMC or z-LLE-AMC (Enzo Life Sciences, Farmingdale, NY) were added to the samples to assess proteasome chymotrypsin-like, trypsin-like and peptidylglutamyl-like activities, respectively (10 μM). The reaction was incubated at 37°C for 30 min (Bz-VGR-AMC and z-LLE-AMC) or 2 h (Suc-LLVY-AMC) and fluorescence monitored at 360 (excitation)/460 (emission) on a monochromatic plate reader (Synergy H1; Biotek, Winooski, VT). Protein free blanks were used and an AMC standard curve was produced according to the manufacturer's instructions.

### Antibodies

Primary antibodies included K48 polyubiquitin (1:1000; Millipore #05-1307, Billerica, MA), Rpt6 (1:500; Enzo Life Sciences #PW9265, Farmingdale, NY), Actin (1:1000; Cell Signaling #4967, Danvers, MA), CaMKII phospho-T286 (1:1000; Abcam #2724, Cambridge, MA), CaMKII (1:1000; Abcam #22609, Cambridge, MA), GluR1 phospho-S845 (1:1000; Millipore #AB5849, Billerica, MA) and GluR1 (1:1000; Millipore #AB1504, Billerica, MA). The phosphorylated Rpt6-Serine120 rabbit polyclonal antibody was generated commercially (ProSci, Poway, CA) against a synthetic peptide [NH_2_-CALRND(pS)YTLHK-OH] as described previously (Djakovic et al., 2012).

### Western blotting

Samples (50 μg) were loaded on 7.5% TGX gels, ran through SDS-PAGE and transferred using a Turbo Transfer System (Biorad, Hercules, CA). Membranes were incubated in 3% milk in TBS + 0.1% Tween-20 (blocking buffer) for 1-h at room temperature, followed by overnight incubation in antibody in 3% BSA in TBS + 0.1% Tween-20. Membranes were then washed and incubated in secondary antibody (1:20,000; Millipore #12-348, Billerica, MA, for goat anti-rabbit, Santa Cruz #SC-2005, Dallas, TX, for goat anti-mouse) in blocking buffer for 60 min. Following a final wash, membranes were incubated in enhanced chemiluminescence substrate (SuperSignal West Dura, Thermo, Pittsburgh, PA) for 5 min and images developed using a CCD-camera based system (GBOX Chemi XT-4; Syngene, Frederick, MD) and analyzed using GeneTools software.

### Conditioned fear responses

The activity of each rat was recorded on digital video and the amount of movement determined by frame-by-frame changes in pixels using FreezeScan 1.0 software (CleverSys, Reston, VA). The automatic scoring parameters are chosen such that the scored activity matches hand-scoring methods previously used in our lab to measure freezing. Freezing detection parameters were as follows (noise filtering radius = 1, Inter-frame motion = 400 pixels, automata parameters-freeze = N-25, M-22; move = N-10, M-8) as previously described (Parsons et al., 2010).

### Statistical analyses

For quantitative protein assays, mean pixel density was calculated for each sample, normalized to actin and taken as a percentage of the control group. For proteasome activity assays, each raw fluorescence reading was standardized to the generated AMC standard curve for that plate and taken as a percentage of the control group. Statistical outliers were determined by SPSS using the “Explore-outliers” function. Data was analyzed using Analysis of Variance (ANOVA), Fisher Least Significant Difference (LSD) *post-hoc* tests and by pairwise *t*-tests where appropriate.

## Results

We first wanted to know if proteasome catalytic activity s increased in the amygdala following fear conditioning. To test this, we trained rats to auditory fear conditioning and then measured proteasome activity in the lysates using an *in vitro* proteasome activity assay (Lopez-Salon et al., 2001; Ehlers, 2003; Upadhya et al., 2006; Bingol et al., 2010). We did not find a main effect for time after conditioning for proteasome activity [*F*_(4, 45)_ = 1.401, *p* = 0.249], but we did for total Rpt6 [*F*_(4, 46)_ = 4.253, *p* < 0.01] and K48 polubiquitination levels [*F*_(4, 46)_ = 3.132, *p* < 0.05]. To determine whether there transient increases in proteasome activity, we did pairwise comparisons for each group against the homecage control. We found that proteasome chymotrypsin-like activity, the predominant type of catalytic activity mediated by the proteasome, gradually increased following learning, peaking at 4 h [*t*_(45)_ = 2.144, *p* < 0.05; Figure 1A) and returning to baseline by 6 h [*t*_(45)_ = 0.843, *p* = 0.404]. This increase at 4 h was not due to an increase in the amount of the proteasome regulatory subunit Rpt6 [*t*_(46)_ = 1.298, *p* > 0.05], but Rpt6 levels were transiently increased at 1 h [*t*_(46)_ = 3.231, *p* < 0.01; Figure 1B] a time at which proteasome activity was marginally higher than control animals [*t*_(45)_ = 1.662, *p* = 0.103]. Protein polyubiquitination levels were increased at 1 h [*t*_(46)_ = 2.507, *p* < 0.05] and 4 h [*t*_(46)_ = 3.179, *p* < 0.01], but not 6 h [*t*_(46)_ = 1.781, *p* = 0.082], relative to controls (Figure 1C), consistent with previous findings (Jarome et al., 2011). This shows that protein polyubiquitination levels peaked before proteasome activity, and rapidly returned to baseline following the peak in proteasome activity. This is consistent with ubiquitin acting as the “tag” for degradation and the proteasome acting as the catalytic structure that degrades tagged proteins. These results suggest that increases in proteasome activity accompany increases in protein polyubiquitination in the amygdala following fear conditioning.

To be sure that the observed increases in proteasome chymotrypsin-like activity 4 h after fear conditioning were specific to learning of the auditory cue—footshock association, we exposed a separate group of rats to control treatments in which the auditory cue or footshock were presented individually and compared proteasome activity in these animals with that from animals who received pairings of the auditory cue and footshock (Figure 2A). During the training session, a mixed variable ANOVA revealed a main effect for time [*F*_(1, 20)_ = 682.513, *p* < 0.001], group [*F*_(1, 20)_ = 327.576, *p* < 0.001] and a time by group interaction [*F*_(1, 20)_ = 483.759, *p* < 0.001]. These results indicate that the animals that received pairings of the auditory cue with the footshock during training showed significantly higher freezing behavior than the white noise alone control, suggesting that they learned an association between the auditory cue and the footshock. We next examined learning-specific changes in proteasome activity in the amygdala following fear conditioning. We did not find main effects for condition for proteasome chymotrypsin [*F*_(3, 37)_ = 1.572, *p* = 0.213] and peptidylglutamyl-peptide hydrolyzing activities [*F*_(3, 37)_ = 0.819, *p* = 0.492], but we did observe a strong trend for a significant effect for proteasome trypsin activity [*F*_(3, 36)_ = 2.779, *p* = 0.055]. To determine if there were learning-specific increases in proteasome activity, we did pairwise comparisons with the trained group against the homecage, SK and WN controls. We found that proteasome activity was significantly increased in the animals that received explicit pairings of the auditory cue and footshock relative to the three associative control groups [*t*_(37)_ = 2.131, *p* < 0.05; Figure 2C]. Additionally, we observed a similar increase in proteasome trypsin-like activity [*t*_(36)_ = 2.390, *p* < 0.05] but a non-significant increase in proteasome peptidylglutamyl-peptide hydrolyzing-like [*t*_(37)_ = 1.528, *p* = 0.135] activity (Figures 2D,E). However, all three types of proteasome activity positively correlated with each other (*r* = 0.884, *p* < 0.01, chymotrypsin with peptidylglutamyl; *r* = 0.735, *p* < 0.01, chymotrypsin with trypsin; *r* = 0.798, *p* < 0.01, peptidylglutamyl with trypsin), suggesting that fear conditioning lead to increases in all three types of proteasome activity in the amygdala. These results suggest that proteasome activity is increased in the amygdala in a learning-dependent manner 4 h after fear conditioning.

Increases in proteasome activity are regulated through phosphorylation of the ATPase subunit Rpt6 of the 19S proteasome (Mabb and Ehlers, 2010). To test this whether proteasome 19S subunits become phosphorylated following behavioral training *in vivo*, we commercially generated a phospho-specific antibody for Rpt6-S120 (Djakovic et al., 2012) and probed amygdala tissue from fear conditioned animals using standard western blotting. We found a strong trend for an effect of group for Rpt6-S120 phosphorylation [*F*_(3, 35)_ = 2.274, *p* = 0.059] and K48 polyubiquitination levels [*F*_(3, 33)_ = 2.821, *p* = 0.054], but not total Rpt6 levels [*F*_(3, 33)_ = 0.582, *p* = 0.631]. To determine if there were learning-specific increases in Rpt6-S120 phosphorylation, total Rpt6 and K48 polyubiquitination levels, we did pairwise comparisons with the trained group against the homecage, SK and WN controls. We found significant increases in phosphorylated Rpt6-S120 in the amygdala of animals exposed to explicit pairings of the auditory cue with the footshock relative to the three associative control groups [*t*_(35)_ = 2.847, *p* < 0.01; Figure 2F]. Furthermore, there were no differences in total Rpt6 between groups [*t*_(33)_ = 0.775, *p* = 0.444; Figure 2G] at this timepoint, but conditioned animals did show greater levels of polyubiquitinated proteins than did controls [*t*_(33)_ = 2.690, *p* < 0.05; Figure 2H]. These results suggest that the learning-induced increases in proteasome activity in the amygdala are related to increased phosphorylation of Rpt6-S120, and suggests for the first time that fear conditioning transiently modifies a proteasome regulatory subunit.

**Figure 3:**
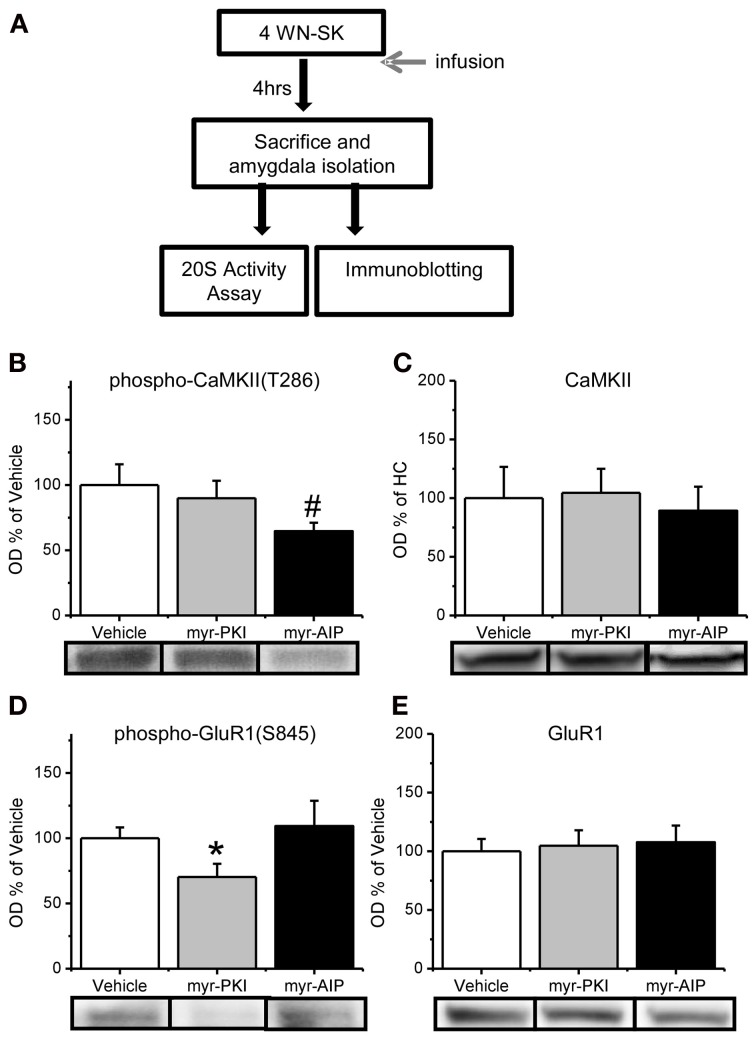
**Reduction in CaMKII or PKA activity in the amygdala. (A)** Rats received infusions of a PKA inhibitor (myr-PKI), CaMKII inhibitor (myr-AIP) or vehicle immediately after fear conditioning and amygdala tissue was collected 4 h later (*n* = 8–10 per group). **(B–C)** Inhibiting CaMKII, but not PKA, tended to reduce the phosphorylation of CaMKII-T286 **(B)** without affecting total CaMKII levels **(C)**. **(D–E)** Inhibiting PKA, but not CaMKII, reduced phosphorylation of GluR1-S845 **(D)** without affecting total GluR1 levels **(E)**. ^*^p < 0.05 from Vehicle and myr-AIP. ^#^*P* = 0.06 from Vehicle and myr-PKI.

We next tested whether CaMKII and PKA regulates the increase in proteasome activity and Rpt6-S120 phosphorylation observed in the amygdala 4 h after fear conditioning (Figure 3A). We trained animals with auditory fear conditioning and microinfused vehicle or myristoylated peptides into the amygdala to specifically block PKA (myr-PKI) or CaMKII (myr-AIP) activity and then collected amygdala lysates 4 h later. We first confirmed the effect of these manipulations on CaMKII and PKA activity in the amygdala by probing amygdala tissue with antibodies for phosphorylated CaMKII-T286 and phosphorylated GluR1-S845, a PKA target site. We did not find a main effect for drug for phosphorylated CaMKII-T286 [*F*_(2, 27)_ = 2.088, *p* = 0.143], total CaMKII [*F*_(2, 26)_ = 0.109, *p* = 0.897], phosphorylated GluR1-S845 [*F*_(2, 27)_ = 2.255, *p* = 0.124] or total GluR1 [*F*_(2, 27)_ = 0.091, *p* = 0.913]. To determine whether the drugs selectively affected their intended molecule, we did pairwise comparisons. We found that the CaMKII inhibitor tended to reduce phosphorylated CaMKII-T286 relative to the vehicle and PKI group [*t*_(27)_ = 1.964, *p* = 0.06; Figure 3B] without any effect on total CaMKII levels [*t*_(26)_ = 0.325, *p* = 0.748; Figure 3C]. Conversely, the PKA inhibitor reduced phosphorylated GluR1-S845 relative to the vehicle and AIP groups [*t*_(27)_ = 2.066, *p* < 0.05; Figure 3D] without affecting total GluR1 [*t*_(27)_ = 0.20, *p* = 0.740; Figure 3E]. These results suggest that our manipulations were effective at reducing either CaMKII or PKA activity in the amygdala.

**Figure 4:**
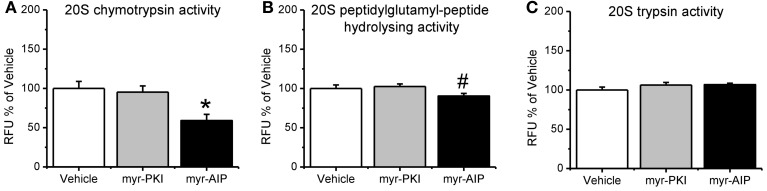
**CaMKII, but not PKA, regulates increases in proteasome activity following fear conditioning.** Rats received infusions of a PKA inhibitor (myr-PKI), CaMKII inhibitor (myr-AIP) or vehicle immediately after fear conditioning and amygdala tissue was collected 4 h later (*n* = 8–10 per group). **(A)** Inhibiting PKA had no effect on proteasome activity, while inhibiting CaMKII significantly reduced proteasome chymotrypsin-like activity relative to vehicle infused trained rats. **(B)** Inhibiting CaMKII, but not PKA, reduced proteasome peptidylglutamyl-peptide hydrolyzing-like activity. **(C)** Neither inhibitor altered proteasome trypsin-like activity. ^*^p < 0.05 from vehicle. ^#^*P* = 0.07 from vehicle.

Next, we tested if the manipulations in CaMKII and PKA altered proteasome activity in the amygdala following fear conditioning. Results indicated a main effect for drug [*F*_(2, 24)_ = 3.330, *p* = 0.053]. We found that inhibiting PKA phosphorylation had no effect on proteasome chymotrypsin-like activity; however, blocking CaMKII activity significantly reduced proteasome activity relative to vehicle infused controls (Figure 4A). Additionally, similar results were found for proteasome peptidylglutamyl-peptide hydrolyzing-like activity [*F*_(2, 27)_ = 2.881, *p* = 0.073; Figure 4B], though neither inhibitor altered proteasome trypsin-like activity [*F*_(2, 27)_ = 1.879, *p* = 0.172; Figure 4C]. These results strongly suggest that CaMKII, but not PKA, is an important regulator of proteasome activity following fear conditioning.

**Figure 5:**
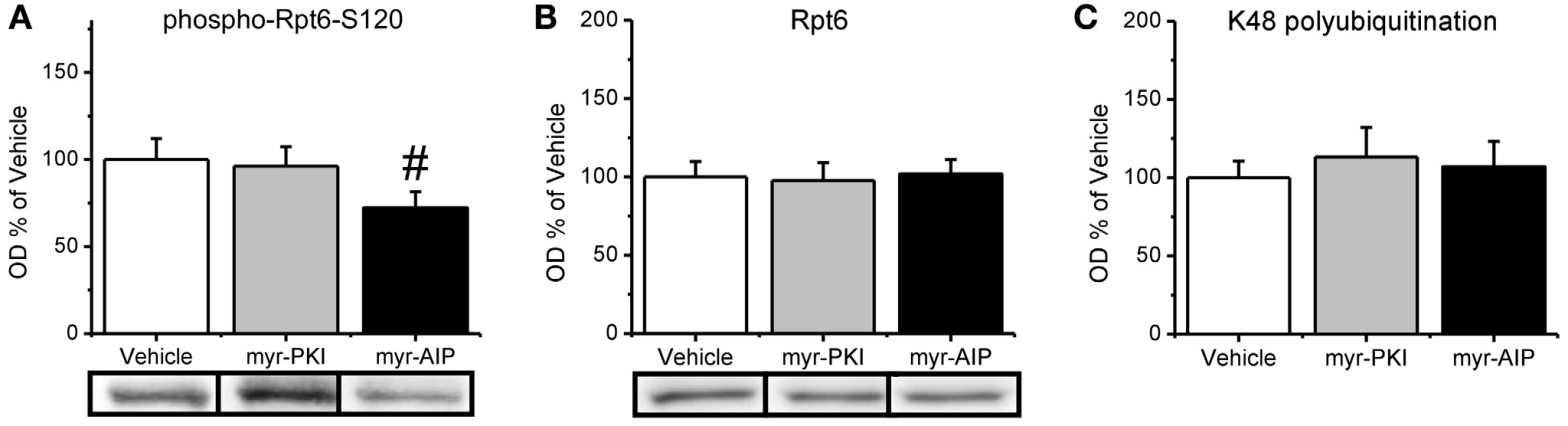
**CaMKII, but not PKA, may be important in the regulation of increases in the phosphorylation of Rpt6-S120 following fear conditioning.** Rats received infusions of a PKA inhibitor (myr-PKI), CaMKII inhibitor (myr-AIP) or vehicle immediately after fear conditioning and amygdala tissue was collected 4 h later (*n* = 8–10 per group). **(A)** Inhibiting CaMKII, but not PKA, tended to decrease Rpt6-S120 phosphorylation in the amygdala following fear conditioning. **(B)** There were no changes in Rpt6 levels. **(C)** None of the drug manipulations reduced learning-induced increases in protein polyubiquitination. ^#^*p* = 0.07 from Vehicle and myr-PKI.

We found that CaMKII, but not PKA, regulates increases in proteasome activity following fear conditioning. Since both CaMKII and PKA can regulate Rpt6-phosphorylation-dependent changes in proteasome activity *in vitro*, this suggests then that manipulation of CaMKII, but not PKA, should reduce the phosphorylation of Rpt6-S120 in the amygdala of fear conditioned rats. To test this we probed our samples with our phospho-Rpt6-S120 antibody (Figure 5A). We did not find a main effect for drug for phosphor-Rpt6-S120 [*F*_(2, 26)_ = 1.799, *p* = 0.185], total Rpt6 [*F*_(2, 27)_ = 0.040, *p* = 0.961] or K48 polyubiquitination [*F*_(2, 25)_ = 0.176, *p* = 0.839]. To determine if there was a selective effect of the CaMKII inhibitor, we did pairwise comparisons with the CaMKII inhibitor group against the vehicle and PKA inhibitor groups. We found that the CaMKII inhibitor resulted in a trend for reduced phosphorylation of Rpt6-S120 relative to the vehicle and PKA inhibitor groups [*t*_(26)_ = 1.890, *p* = 0.07] without effecting total Rpt6 levels [*t*_(27)_ = 0.238, *p* = 0.841; Figure 5B] or protein polyubiquitination [*t*_(25)_ = 0.024, *p* = 0.981; Figure 5C]. Together, these results strongly suggest that CaMKII, but not PKA, is an important regulator of Rpt6-S120 phosphorylation and increases in proteasome activity following fear conditioning.

## Discussion

It has been widely supported that the formation of long-term fear memories requires increases in gene transcription and *de novo* protein synthesis in the amygdala (Bailey et al., 1999; Schafe and LeDoux, 2000; Parsons et al., 2006; Jarome et al., 2011) and increases in protein synthesis have been reported in the amygdala following fear conditioning (Hoeffer et al., 2011), but only recently has the role of protein degradation in memory formation and stability been examined (Yeh et al., 2006; Artinian et al., 2008; Lee, 2008; Lee et al., 2008; Jarome et al., 2011; Monopoli et al., 2011; Rodriguez-Ortiz et al., 2011). We have shown that fear conditioning increases degradation-specific polyubiquitination in the amygdala, which is NMDA receptor-dependent and mirrors increased translational regulation, and blocking proteasome activity in the amygdala following fear conditioning significantly impairs long-term memory formation (Jarome et al., 2011). While we demonstrated that the increases in protein polyubiquitination were dependent on NMDA receptor activity, it is unclear how protein degradation is regulated during memory formation downstream of NMDA receptors. Additionally, it is unknown how the proteasome's activity is altered or regulated following learning. Here, for the first time, we demonstrate that fear conditioning increases the phosphorylation of proteasome regulatory subunit Rpt6 at Serine-120 and proteasome catalytic activity, and that CaMKII, but not PKA, regulates increases in Rpt6-S120 phosphorylation and proteasome activity during memory formation. This is consistent with *in vitro* work demonstrating that CaMKII can regulate the proteasome (Djakovic et al., 2009; Bingol et al., 2010; Djakovic et al., 2012; Hamilton et al., 2012). Interestingly, *in vitro* work has shown that PKA can regulate the proteasome (Upadhya et al., 2006; Zhang et al., 2007), but our work suggests that PKA-mediated proteasome regulation is not necessary for memory formation. This suggests a novel signaling pathway through which CaMKII mediates memory formation in the amygdala, by phosphorylating the proteasome regulatory subunit Rpt6 which results in increases in proteasome activity necessary for long-term memory formation (Jarome and Helmstetter, 2013). Whether phosphorylation of Rpt6-S120 is necessary for increases in proteasome activity and long-term memory storage following learning will be of interest in future research.

While a number of studies have implicated the ubiquitin-proteasome system in various types of synaptic plasticity (Ehlers, 2003; Mabb and Ehlers, 2010), very little is known about how the proteasome is regulated during increased levels of synaptic activity. Activation of NMDA receptors has been shown to induce the movement of proteasomes into dendritic spines and increase their activity (Bingol and Schuman, 2006). More recently, CaMKII has been shown to act as a downstream regulator of proteasome activity, acting as a scaffold to recruit proteasomes to dendritic spines where it can then increase their activity by phosphorylation of the proteasome regulatory subunit Rpt6 at Serine-120 (Bingol et al., 2010). Consistent with this, the phosphorylation state of Rpt6 mimics changes in synaptic strength that normally occur from chronic stimulation or inhibition of cultured hippocampal neurons (Djakovic et al., 2012) and promotes the growth of new dendritic spine *in vitro* (Hamilton et al., 2012). It is unclear though if CaMKII regulates proteasome activity *in vivo*. Here, we found that specifically inhibiting CaMKII activity with the myristoylated peptide AIP significantly reduced conditioning-induced enhancements in the phosphorylation of Rpt6-S120 and proteasome activity in the amygdala of rats. This suggests that CaMKII likely regulates proteasome activity through a Rpt6-S120-dependent mechanism both *in vitro* and *in vivo*. Interestingly, we found that PKA, which is also known to regulate Rpt6-S120 phosphorylation and proteasome activity *in vitro*, does not seem to be critical for the regulation of proteasome subunit phosphorylation or proteasome activity during memory formation. Collectively, these results suggest that while both CaMKII and PKA can regulate proteasome activity *in vitro*, CaMKII, but not PKA, is may be the primary regulator of proteasome activity *in vivo*.

Numerous studies have shown that proteasome inhibitors applied into various brain regions can alter long-term memory formation (Lopez-Salon et al., 2001; Yeh et al., 2006; Artinian et al., 2008; Lee et al., 2008; Jarome et al., 2011), suggesting that functional proteasome activity is critical for memory formation. However, it is currently unknown if proteasome activity is altered as a function of learning or if basal proteasome activity is sufficient to regulate the increased demand for protein degradation in the brain. We found that fear conditioning lead to increases in proteasome activity in the amygdala, suggesting that increases in proteasome activity accompany increases in protein polyubiquitination in the amygdala, which is consistent with previous data from the hippocampus (Lopez-Salon et al., 2001). This result suggests that dynamic changes in overall ubiquitin-proteasome activity are critical for fear memory formation in the amygdala. Interestingly, we found that protein polyubiquitination levels returned to baseline immediately after proteasome activity increased. This suggests that while basal proteasome activity may be sufficient to regulate increases in protein degradation early on in the consolidation process, increases in proteasome activity are likely necessary to regulate the enhancements in protein degradation during memory formation. These results suggest that proteasome inhibitors likely impair memory by preventing increases in proteasome catalytic activity following behavioral training.

In the present study we found increases in proteasome activity as a function of learning, however, it is unknown what functional role these increases in proteasome activity serve. One possibility is that they regulate increases in gene transcription and postsynaptic density remodeling (Kaang and Choi, 2012; Jarome and Helmstetter, 2013). Consistent with this, the proteasome has been shown to target transcriptional and translational repressors and synaptic scaffolding proteins during the consolidation process (Lopez-Salon et al., 2001; Jarome et al., 2011). However, very few targets of the proteasome have been identified following behavioral training and no study has directly tested if proteasome activity is critical for changes in transcription/translation and postsynaptic remodeling during the consolidation process. Future research will need to more directly examine these potential roles of the ubiquitin-proteasome in long-term memory formation.

In conclusion, our results indicate that learning dynamically alters proteasome phosphorylation and activity and suggests a novel role for CaMKII during memory formation. We found that pharmacologically inhibiting CaMKII significantly reduced both phosphorylation of Rpt6-S120 and proteasome activity following fear conditioning. Interestingly, inhibiting PKA, which is also known to regulate Rpt6-S120 phosphorylation and proteasome activity *in vitro*, had no effect on the increases in Rpt6-S120 phosphorylation and proteasome activity seen during the memory consolidation period. Collectively, these results suggest that in addition to its possible regulation of transcription and translation, CaMKII also regulates changes in protein degradation during memory formation. In the latter, CaMKII regulates phosphorylation of proteasome regulatory subunit Rpt6 at Serine-120 which likely mediates increases in proteasome activity following fear conditioning, indicating a novel signaling pathway by which CaMKII regulates memory formation in the amygdala and suggests that CaMKII regulates proteasome activity both *in vitro* and *in vivo*.

### Conflict of interest statement

The authors declare that the research was conducted in the absence of any commercial or financial relationships that could be construed as a potential conflict of interest.
